# Supplementary material for: Help seeking behavior by women experiencing intimate partner violence in india: A machine learning approach to identifying risk factors
Source: PLoS One. 2022 Feb 3;17(2):e0262538. doi: 10.1371/journal.pone.0262538 (PMC8813002; doi:10.1371/journal.pone.0262538)
Supplement: S1 Fig — (DOCX) [file pone.0262538.s001.docx]

S1: Flowchart of the Iterative Thematic Analysis process

No new themes identified in more than 3 consecutive iterations/No new variables in consecutive iteration

L1-Logistic regression/ lasso

L2-Logistic regression (with variables that had non-zero coefficient value in lasso)

Select list of variables with coefficient value higher than knee point

Researcher 1: Categorize the variables into different themes

Researcher 2: Categorize the variables into different themes

Discuss and finalize final categories and coding for each variable

Exclude the group of variables corresponding to the theme with the variable that has the highest coefficient value

L1-Logistic regression/ lasso

L2-Logistic regression/ridge

Researcher 1: Categorize the variables into different themes

Researcher 2: Categorize the variables into different themes

Discuss and finalize final categories and coding for each variable

List includes one or more variables corresponding to a theme already excluded - exclude these identified variables

List of variables with coefficient value higher than knee point

List does not include any variable corresponding to a theme already excluded

STOP

Figure adapted from: Raj A, Dehingia N, Singh A, McDougal L, McAuley J. Application of machine learning to understand child marriage in India. SSM-population health. 2020;12:100687
