# Supplementary material for: Help seeking behavior by women experiencing intimate partner violence in india: A machine learning approach to identifying risk factors
Source: PLoS One. 2022 Feb 3;17(2):e0262538. doi: 10.1371/journal.pone.0262538 (PMC8813002; doi:10.1371/journal.pone.0262538)
Supplement: S1 Table — (DOCX) [file pone.0262538.s002.docx]

S1: Characteristics of all women included in the sample

|  | Total sample included in the analysis (N = 19,468) |
| --- | --- |
| **Characteristics** | **Wtd. %/Mean** |
| Sources of help^1^ |  |
| Own family | 9.2% |
| Husband/partner's family | 5.0% |
| Neighbor | 1.7% |
| Friend | 1.9% |
| Social service organization | 0.1% |
| Religious leader | 0.3% |
| Doctor | 0.2% |
| Lawyer | 0.2% |
| Police | 0.6% |
| Other | 0.3% |
|  |  |
| Age | 33.8 |
| Literate | 51.2% |
| Education |  |
| None | 43.1% |
| Primary | 17.3% |
| Secondary | 35.2% |
| Higher | 4.5% |
| Household wealth quintile: |  |
| Poorest | 24.0% |
| Poorer | 23.8% |
| Middle | 21.9% |
| Richer | 18.3% |
| Richest | 12.0% |
| Religion |  |
| Muslim | 12.5% |
| Hindu and Others | 87.5% |
| Caste |  |
| SC/ST | 35.3% |
| OBC | 47.2% |
| Other caste/General | 17.5% |
| Place of residence: |  |
| Rural | 71.8% |
| Urban | 28.2% |
| Region of residence |  |
| North | 9.3% |
| West | 10.3% |
| South | 26.3% |
| Northeast | 2.9% |
| East | 26.9% |
| Central | 24.3% |
